# Predicting Placebo Responses Using EEG and Deep Convolutional Neural Networks: Correlations with Clinical Data Across Three Independent Datasets

**DOI:** 10.1007/s12021-025-09725-6

**Published:** 2025-05-19

**Authors:** Mariam Khayretdinova, Polina Pshonkovskaya, Ilya Zakharov, Timothy Adamovich, Andrey Kiryasov, Andrey Zhdanov, Alexey Shovkun

**Affiliations:** 1 Brainify.AI, 101 Americas Avenue, 3 Floor, NY City, NY 10013 USA; 2 Centre for Mathematical Sciences, University of Cambridge, Wilberforce Rd, Cambridge, CB3 0 WA UK

**Keywords:** Placebo response, Resting-state EEG, DCNN, Clinical features, Machine learning, Predictive modeling

## Abstract

Identifying likely placebo responders can help design more efficient clinical trials by stratifying participants, reducing sample size requirements, and enhancing the detection of true drug effects. In response to this need, we developed a deep convolutional neural network (DCNN) model using resting-state EEG data from the EMBARC study, achieving a balanced accuracy of 69% in predicting placebo responses in patients with major depressive disorder (MDD). We then applied this model to two additional datasets, LEMON and CAN-BIND—which did not include placebo groups—to investigate potential relationships between the model's predictions and various clinical features in independent samples. Notably, the model's predictions correlated with factors previously linked to placebo response in MDD, including age, extraversion, and cognitive processing speed. These findings highlight several factors associated with placebo susceptibility, offering insights that could guide more efficient clinical trial designs. Future research should explore the broader applicability of such predictive models across different medical conditions, and replicate the current EEG-based model of placebo response in independent samples.

**Supplementary Information:**

The online version contains supplementary material available at 10.1007/s12021-025-09725-6.

## Introduction

The placebo effect, a well-documented psychobiological phenomenon, is characterized by symptomatic improvement following the administration of an inert treatment, owing to the patient’s belief in the treatment’s efficacy (Kelley et al. 2012). It essentially illustrates that the anticipation of benefit can itself trigger health improvements, independent of the treatment's active components (Rutherford and Roose 2013;, Rutherford et al. 2017). The high placebo response rate, observed to be between 30 and 40% in clinical trials for major depressive disorder (MDD), poses significant challenges for the development of new treatments (Furukawa et al. 2016;, Fava et al. 2020). Further complicating matters, placebo response may exhibit features akin to those of active drugs, such as latency periods before effects manifest and a potential for a maximum effect over time (Lasagna et al. 1954).

One of the main concerns is that placebo effects have become a major obstacle during clinical trials since they compete with the potential drug effects, often resulting in high failure rates in randomized controlled trials for MDD treatments. In the context of antidepressant medication, the measure of the active drug’s superiority to placebo — the effect size measured by Cohen's d — is typically around 0.3, indicating a modest difference between medication and placebo in terms of therapeutic efficacy (Fournier et al. 2010; Cipriani et al. 2018). Accurately predicting individuals likely to exhibit a placebo response may enable more effective stratification of clinical trial participants. Specifically, rather than excluding placebo responders from receiving treatment, predicted placebo responsiveness can be incorporated as a covariate or used to define subgroups within statistical analyses. This allows researchers to better control for placebo effects statistically, thus enhancing the ability to differentiate true pharmacological effects of the investigational drug from placebo effects, ultimately increasing the statistical power and reliability of clinical trial outcomes.

Placebo effects could be influenced by a variety of underlying neurobiological, genetic, and psychological mechanisms that are common across different conditions. Research into placebo effects reveals variability across different demographic groups. For instance, meta-analyses by Vambheim et al. (Vambheim and Flaten 2017) and Weimer et al. (Weimer et al. 2015) have examined sex and age differences in placebo responses, suggesting that males may exhibit a stronger placebo response than females, possibly due to being particularly influenced by verbal suggestions. However, these sex differences are observed across a limited number of medical conditions and require further examination. Age also has been associated with variability in placebo responses. Specifically, Weimer et al. (Weimer et al. 2015) reported that younger individuals may exhibit higher placebo response rates in psychiatric and pain trials. This is likely primarily attributed to their lack of negative treatment experiences and treatment-naïve status. Moreover, research utilizing a framework focused on Big Five personality dimensions has emphasized that specific personality characteristics might affect susceptibility to placebo effects. Among the traits examined, extraversion has consistently emerged as a significant predictor of placebo response. Moreover, research by Trivedi et al. (Trivedi et al. 2018) and Ang et al. (Ang et al. 2022) identified specific characteristics linked to higher placebo responses in MDD, including being younger, having quicker cognitive processing, having less anhedonia and no melancholic features, and having no previous history of trauma or abuse.

To understand the neural correlates of the placebo response, brain imaging technologies such as functional magnetic resonance imaging (fMRI) can be used. fMRI has been instrumental for improving our understanding of the neurobiology of placebo response (e.g (Zunhammer et al. 2021;, Tétreault et al. 2016).); however high costs and the need for technical expertise might prevent wide adoption of this technology. A cheaper and more scalable alternative is electroencephalography (EEG). Owing to its high (millisecond) temporal resolution, EEG can capture signatures of fast neural activity during both resting states and cognitive processes (Trivedi et al. 2018; Ang et al. 2022; Trivedi et al. 2016; –Pizzagalli et al. 2018). Trivedi et al. (Trivedi et al. 2016) utilized data from the Establishing Moderators and Biosignatures of Antidepressant Response in Clinical Care (EMBARC) study to develop a predictive index for placebo response, incorporating both clinical and EEG features. To achieve this, they employed an elastic net method, a type of penalized regression technique that is particularly useful in scenarios where there are many candidate predictor variables, and the model seeks to enhance prediction accuracy while managing model complexity.

More recent studies have employed other machine learning methods to improve the accuracy of detection of placebo responders. Wu and colleagues (Wu et al. 2020)developed a machine learning algorithm, SELSER, to predict antidepressant and placebo response using resting-state electroencephalography (rsEEG) data. A significant finding was that out of all the band powers, only alpha frequency from the resting eyes open (REO) condition was a significant predictor of treatment outcomes with sertraline, achieving a Pearson's correlation of 0.60. Recently, Oakley et al. (Oakley et al. 2023) developed Random Forest (RF) and Support Vector Machine (SVM) Classifiers for the task of response prediction to Sertraline and Placebo in the EMBARC dataset. The SVM model achieved a classification accuracy of 83%, with a sensitivity of 75.4% and a specificity of 88.1%. The RF model, which uses an ensemble of decision trees to improve prediction accuracy and robustness, also performed well, achieving an accuracy of 78.9%, with a sensitivity of 70.2% and a specificity of 84.4%.

Traditional machine learning methods for EEG signal classification typically rely on manual feature extraction, feature selection, and conventional classification algorithms. However, recent advancements in deep neural networks (DNNs), particularly deep convolutional neural networks (CNNs) (Durstewitz et al. 2018), have demonstrated significant potential in the classification of psychiatric disorders, including dementia, attention deficit hyperactivity disorder (ADHD), schizophrenia, depression and others (Wang et al. 2022). A notable advantage of DNNs, and specifically deep CNNs, lies in their capacity to process raw high-dimensional data directly, thereby eliminating the need for manual feature extraction, a limitation of traditional machine learning approaches. DCNNs have been applied to EEG classification by converting signals into images (Krizhevsky et al. 2017). Wang et al. (Wang et al. 2022) performed the classification task using dCNNs and achieved accuracy levels of up to 87.71% in distinguishing between normal EEG samples and those from patients with psychiatric disorders (depression and schizophrenia).

In this study, we utilized a dCNN model to predict placebo responses, leveraging EEG data from the EMBARC study. The deep CNN model was chosen for its ability to capture temporal dependencies in EEG signals, improving the accuracy of predictions compared to traditional machine learning approaches that treat data points independently. Our model and earlier attempts at predicting placebo responders were developed and/or validated using the EMBARC dataset, reflecting the limited availability of large-scale placebo EEG data. To address this data scarcity, we incorporated data augmentation techniques, including re-referencing and Gaussian noise injection, enabling the model to learn from a broader dataset by generating additional synthetic data from existing samples. Our main hypothesis was that the integration of behaviorally rich metadata, alongside EEG data, would reveal significant correlations between the model’s predictions based on neural data and participants'demographic, psychological, and cognitive characteristics. We hypothesized that analyses on independent datasets would support the predictive performance of our model and reveal consistent associations between the placebo responses and the extensive demographic and psychological variables available, providing valuable insights for refining clinical trials and optimizing drug development.

## Methods

### Datasets Characteristics

Our model development was based on data derived from the placebo arm of the EMBARC (Trivedi et al. 2016) study, which included 141 participants. Subsequently, model robustness was evaluated by checking for stability and reliability when applied to the data it was trained on. Due to the lack of publicly accessible EEG datasets from clinical trials that include placebo response data, we adopted an indirect method by investigating participants’ characteristics linked to placebo response. Specifically, we analyzed whether the responsiveness to placebo treatment predicted by our model (a binary variable, classifying each participant as either placebo responder or non-responder) covaried with participant metadata such as age, sex, personality traits, and symptom severity. To this end, we selected the CAN-BIND (MacQueen et al. 2019) and LEMON (Babayan et al. 2019) datasets for correlational analysis due to their comprehensive clinical, behavioral and physiological metadata. We hypothesized that if our model's predictions correlated with key participant metadata in an external dataset, this would indicate the model's potential for further development. For detailed demographic and clinical characteristics of these datasets, please see Table 1**.**

**Table 1:** Overview of EEG datasets utilized for placebo response prediction model development

Dataset Name	N Total Participants males/females	N Placebo males/females	Age Range	Questionnaire for Depression	Conditions	EEG Procedure	Number of Channels
EMBARC	322/182	141/89	18–65 (mean = 37.64, SD = 12.9)	HDRS- 17	MDD	Resting-state, 2-min EC and EO conditions	72
CAN-BIND	180/76	N/A	18–60 (mean = 34.78, SD = 11.71)	MADRS	MDD	8-min of resting-state EC	64
LEMON	227/119	N/A	20–35, 59–77 (mean = 25.1 SD = 3.1; mean = 67.7, SD = 4.7)	N/A	Healthy and depressed	8-min of EC and 8-min of EO	62

Abbreviations: *EMBARC* Establishing moderators and biosignatures of antidepressant response in clinical care, *CAN-BIND* The Canadian Biomarker Integration Network in Depression, *LEMON* Leipzig Study for Mind–Body-Emotion Interactions, *EC* eyes closed, *EO* eyes opened, *MDD* major depressive disorder

### EEG Preprocessing Pipeline

We implemented a standardized preprocessing protocol to clean the raw EEG recordings across the LEMON, EMBARC, and CAN-BIND datasets. We implemented a preprocessing protocol to clean the raw EEG recordings from the EMBARC dataset. First, a bandpass filter ranging from 0.5 to 100 Hz was applied to the EEG data, complemented by notch filters at 50 and 60 Hz. To isolate and subtract eye-movement-related artifacts, we employed an Independent Component Analysis (ICA)-based ocular correction using the MNE toolbox (Gramfort et al. 2014). After correcting for ocular activity, we identified and removed portions of the recording in which any of the following artifacts were detected:

Muscle activity (75–95 Hz range).

Extreme voltage fluctuations (> 200 µV).

Abrupt signal transitions (> 2 standard deviations above the recording mean baseline).

High kurtosis (> 4 z-score), indicating signal spikiness.

Persistent eye blinks.

Electrode bridging.

The data were then segmented into 16-s intervals with a 15-s overlap and resampled at 125 Hz. All segments containing any artifacts were excluded from the sample.

We utilized 19 channels corresponding to the International 10–20 system. Data from the MGH site were recorded using a Geodesic Sensor Net (GSN) with 128 electrodes that do not directly correspond to the 10–20 system; therefore, the closest electrodes from the GSN were used as substitutes (see Appendix 1 for the Electrode Conversion Table).

Each segment had an overall shape of [19 channels × 2,000 time points]. The total number of segments was approximately 32,000 for the baseline model and approximately 7,000 for the fine-tuned model.

Several data augmentation techniques were employed:

Re-referencing to a random channel.

Addition of Gaussian noise (mean = 0; standard deviation randomly selected between 0 and 0.2).

Amplitude amplification of a random channel, up to 1.2 times the original amplitude.

Horizontal flipping of the data.

Random horizontal scaling: the original signal was compressed along the x-axis and zero-padded back to the original size.

### DCNN Description and Training

**Fig. 1:**
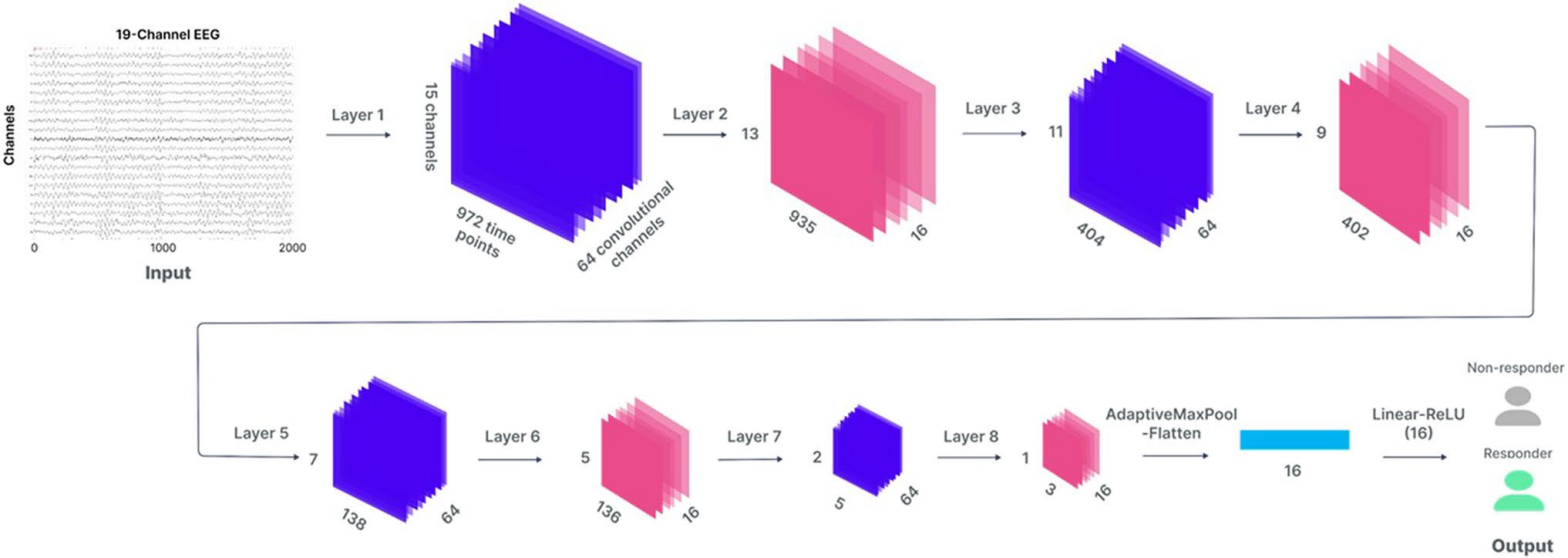
The architecture of the deep CNN model used for placebo response classification. The model consists of eight distinct blocks, beginning with an input layer (illustrating a 2000-point time series on the x-axis). The y-axis represents the number of channels, and the z-axis denotes the number of convolutional channels. The architecture processes the input through a series of convolutional channels (Conv), each followed by batch normalization (BN) and rectified linear unit (ReLU) activations. Convolutional channels progressively reduce the input size and extract meaningful features across time points. Pooling operations (AdaptiveMaxPool) further reduce the spatial dimensions before the final fully connected layers, which output a binary classification: placebo responder or non-responder

We utilized a dCNN as the foundation for our model (see Fig. 1). DCNNs are particularly adept at recognizing patterns in data, similar to how they have revolutionized image recognition tasks by identifying complex patterns in pictures. The input signal undergoes convolution, resulting in feature extraction. Batch normalization was then applied to normalize the data and improve training efficiency (Ioffe and Szegedy 2015). The Rectified Linear Unit (ReLU) activation function was used to introduce nonlinearity and enhance the model's ability to handle noisy input data (Agarap 2018). After the convolutional blocks, global average pooling was performed to transform the multidimensional tensor into a one-dimensional vector. Next, a linear layer was subsequently applied to this vector to generate logit predictions.

To maximize possible future applications, the model was trained on 19-channel, 8-s segments of raw EEG data with 250 Hz sampling rate. We utilized 19 channels of the International 10–20 system (Fp1/2, F7/8, F3/4, Fz, T7/8, C3/4, Cz, P7/8, P3/4, Pz, O1/2) to ensure maximum applicability of the model for diagnostics and compatibility with a wide range of EEG devices and commonly used electrode layouts.

The training consisted of two main stages. In the first stage, referred to as the'baseline'model in the article, the model was trained on the entire EMBARC dataset to predict remission across both treatment and placebo groups. Remission was defined as a Hamilton Depression Scale (HAM-D- 17) score of ≤ 7, regardless of whether it was triggered by placebo or active drug. In the second stage, the model was fine-tuned to specifically predict placebo responders by constraining the training to only include subjects who received a placebo. Each model was trained separately, and fine-tuning was performed only after the training of the baseline model was completed. During fine-tuning, only the parameters of layer L8 and the classifier layer were updated while all other model weights were frozen, allowing the model to specialize in predicting placebo responders by adjusting higher-level features without altering the lower-level representations learned during initial training.

### DCNN Model Validation

To evaluate the model's performance and prevent overfitting, a fivefold cross-subject cross-validation approach was employed. The dataset was divided into 5 equal parts, with each part serving as a fold. In each iteration, 3/5 of the data were used for training, 1/5 for validation during training, and 1/5 for final testing. Importantly, all segments belonging to the same subject were retained within the same fold to prevent data leakage and ensure subject-independent evaluation.

During training, the model's performance was monitored on the validation set to guide hyperparameter tuning and prevent overfitting. An"early stopping"technique was implemented, halting training after 20 epochs if there was no improvement in the model's performance on the validation set. This approach helped prevent the model from overfitting to the training data by stopping the learning process when further improvements were unlikely.

The use of cross-validation allowed for a robust assessment of the model's generalization capabilities across different subsets of the data. By iterating through all five folds, each subset of the data was used for validation and testing exactly once, providing a comprehensive evaluation of the model's performance.

We grouped all EO and EC session segments from each subject into the same fold, ensuring that the model focused on detecting underlying patterns across different EEG recordings rather than memorizing individual sessions.

### DCNN Model Testing

The performance of the trained model was evaluated using the PyTorch and Catalyst libraries. A batch size of 512 segments was utilized during testing to ensure consistency with the training phase and to optimize computational efficiency.

The testing process employed the same computational environment as during training to maintain consistency and reproducibility. Although optimization parameters such as the learning rate and optimizer are primarily relevant during training, they were kept consistent during testing for uniformity. Specifically, the AdamW optimization algorithm (Loshchilov 2017) was used with a weight decay of 0.1, mirroring the training configuration. The initial learning rate for the base model was set to 3 * 10^–5^, while fine-tuning utilized a learning rate of 1 * 10^–4^.

While the"Reduce on Plateau"scheduler with a patience of ten epochs and a reduction factor of 0.5 was employed during training to adjust the learning rate when the monitored metric ceased improving (Wu and Liu 2023), it was not active during testing since no parameter updates occur in this phase. Similarly, the"early stopping"technique, which halted training after 20 epochs without improvement on the validation set to prevent overfitting, was not applicable during testing.

The testing parameters were consistent across both the baseline and fine-tuned models to facilitate a fair comparison of their predictive performance. The test dataset was entirely separate from the training and validation datasets to prevent data leakage and to provide an unbiased assessment of the model's generalization capabilities.

The model's performance on the test dataset was assessed using evaluation metrics appropriate for binary classification tasks. These included accuracy, precision, recall, F1-score, and the area under the Receiver Operating Characteristic (ROC) curve. The Binary Cross-Entropy with logits loss function was employed to compute the loss between the predicted outputs and the true labels during testing. These metrics provided a comprehensive evaluation of the model's ability to accurately predict remission outcomes in unseen data.

### Model Bootstrapping

To ensure robustness and reliability of the results, we trained twenty models with identical architectures but different fold splits. This approach was employed to minimize any potential influence of data partitioning on the outcomes. Each model was trained using a new random seed for model initialization and training, as well as a unique train-validation-test split.

The final accuracy score was determined through bootstrapping with 1,000 repetitions. At each bootstrapping step, we randomly selected prediction values with replacement from all models for each segment in the data. The predictions were then averaged across models for each segment and session. The accuracy score for the sample was calculated, and the reported accuracy is presented as the mean ± standard deviation of the resulting accuracy distribution.

### Estimation of Spectral Power Effect on Prediction

We employed a mixed-effects linear model to evaluate the relationship between the predictor variable and placebo prediction score, incorporating sex and eye state as interacting factors.

The model was specified as:

$$Prediction \sim \{predictor\} + sex*eye\_state$$

where subject id was treated as a random effect to account for inter-individual variability. The predictors consisted of the absolute spectral power values in four frequency bands—theta (4–8 Hz), alpha (8–13 Hz), lower beta (13–20 Hz), and upper beta (20–30 Hz)—calculated individually for each channel. To ensure the robustness of our results, a bootstrap resampling procedure was applied. Across 1000 iterations, the model was fitted to bootstrapped datasets, generated by resampling participants with replacement while maintaining the original sample size.

The significance was based on the distribution of regression coefficients obtained from the bootstrapped linear models. A regression effect was considered significant if the two-tailed 5% confidence interval of the coefficient distribution did not include zero.

Estimation of the relationship between prediction and potential demographic and behavioural covariates. To identify potential covariates and factors influencing the placebo score, we computed the Pearson correlation coefficient, applying Holm’s correction to account for multiple comparisons.

## Results

### DCNN Model Accuracy

Our dCNN model was developed to address the classification problem of predicting placebo response from EEG data (Table 2). In this binary classification task, the model was tasked with distinguishing between individuals who would exhibit a placebo response and those who would not, based solely on their EEG patterns. The model achieved a 0.72 ROC AUC (std 0.009) and 0.696 (std 0.019) Balanced Accuracy (BAcc) in this classification task, effectively identifying individuals likely to experience placebo effects. In comparison, the model trained on the placebo arm without baseline pre-training achieved accuracy of 0.515 ± 0.004.

**Table 2:** Placebo response model accuracy

Model	Loss (mean ± std)	AUC (mean ± std)	BAcc (mean ± std)	Sensitivity (mean ± std)	Specificity (mean ± std)
Baseline	0.6924 ± 0.0011	0.5562 ± 0.0041	0.5513 ± 0.0068	0.606 ± 0.025	0.511 ± 0.021
Fine-tune	0.649 ± 0.003	0.728 ± 0.01	0.696 ± 0.02	0.6787 ± 0.0281	0.6486 ± 0.0285

Abrviation: *N* Number of samples and its standard deviation, *Loss* The loss value and its standard deviation, *AUC* Area Under the ROC Curve and its standard deviation, *BAcc* Balanced Accuracy and its standard deviation.

### Placebo Response Correlations with EEG Band Power

We performed regression analyses between EEG band power and placebo outcomes. These analyses aim to identify specific frequency bands and electrode locations that may serve as driving predictors for placebo response and to which our model is particularly susceptible.

The six main predictors with the highest absolute regression coefficient include, the values are expressed in a "mean±standard deviation" format: F8 (− 0.1135 ± 0.0071), central Cz (− 0.1099 ± 0.0065), Fz (− 0.1022 ± 0.0064), C3 (− 0.06034 ±0.00739) in theta band (4–8 Hz), F4 (− 0.1059 ± 0.0060), and F3 (− 0.0993 ± 0.0061) in the lower beta-frequency (13–20 Hz) range. There is also a consistent negative effect of sex, with males exhibiting lower predicted placebo response (mean coefficient: –0.12). This suggests significant and robust sex-related differences in underlying brain activation patterns.

### Model Predictions on the External Datasets

To indirectly evaluate the potential demographic and psychological covariates and confounders, we tested for putative relationships between the output prediction of the model and various metadata characteristics of the CAN-BIND and LEMON samples. This is critical to understand the model's application beyond the initial study parameters. For detailed demographic and clinical characteristics of these datasets, please see Table 1**.**

As hypothesized, several participants’ characteristics were associated with placebo response across datasets. In particular, age was negatively associated with placebo response across datasets, suggesting that younger patients might be more susceptible to placebo effects. Such negative correlation between model predictions and age emerged across all three datasets (EMBARC: *r* = − 0.46; *p <* 0.001; CAN-BIND: *r* = − 0.35, *p <* 0.001; LEMON: *r* = − 0.29, *p <* 0.001). Table 3 summarizes correlations between the placebo model predictions and the overlapping metadata.

**Table 3:** Correlations between the placebo model predictions and CAN-BIND and LEMON datasets metadata

Dataset	EMBARC	CAN-BIND	LEMON
Demographic variables			
*Age*	− 0.46***	− 0.35***	− 0.29***
*Sex*	ns	ns	ns
*Education*	ns	ns	ns
Personality variables			
	0.14* (Extraversion)	0.21* (Behavioral Inhibition)	0.22** (Sensation seeking)
		− 0.13* (Self-esteem)	− 0.15* (External Thinking)

* *p < *0.05, **p < 0.005, ***p < 0.001, *ns* not significant. The text in brackets indicates the construct behind the variables, the details of the measurements of the variables in different datasets are presented in Table 1

When considering personality traits, in the LEMON dataset positive correlations emerged with extraversion (*r* = 0.14, *p < *0.05), behavioral inhibition (*r* = 0.21, *p < *0.05), and sensation seeking (*r* = 0.22, *p < *0.005), implying that these traits might be associated with a higher placebo effect. Conversely, self-esteem (*r* = − 0.13, *p < *0.05) and externally oriented thinking (*r* = − 0.15, *p < *0.05) had negative correlations, implying that lower levels of these traits might predict a greater placebo response.

The model predictions exhibited a consistent relationship with reaction times and cognitive test performance for both LEMON and CAN-BIND datasets. The information regarding the cognitive tasks and questionnaires can be found in Table 4. In the LEMON dataset, inverse relationships emerged with various cognitive variables. Specifically, slower reaction times in response to incongruent stimuli (*r* = − 0.27, *p < *0.005) in Flanker task and working memory trials (*r* = − 0.14, *p < *0.05) were associated with lower placebo effect. Furthermore, the model highlighted a propensity for a stronger placebo effect with better logical reasoning (*r* = 0.21, *p < *0.05) and higher scores on the New York Cognition Questionnaire (NYC-Q) (*r* = 0.18, *p < *0.05), which evaluates how well specific statements describe participants'thoughts and the nature of their thought processes. Analysis of the CAN-BIND dataset revealed complementary insights. Both the Symbol Digit Coding Test (*r* = 0.10, *p < *0.05) and Shifting Attention Test (*r* = 0.10, *p < *0.05) showed positive associations between reaction times and placebo response. Interestingly, the Shifting Attention Test's average correct reaction time showed a significant negative correlation (*r* = − 0.26, *p < *0.005), aligning with findings from the Four-Part Continuous Performance Test, both in its fourth part average correct (*r* = − 0.21, *p < *0.05) and incorrect reaction times (*r* = − 0.11, *p < *0.05). These findings from the CAN-BIND dataset further elucidate the relationship between cognitive processing speeds and placebo response in the context of our model.

**Table 4:** Overview of significant correlations between cognitive assessments and questionnaires and placebo response

Dataset	Task/Scale	Characteristics	Correlation with Placebo
CAN-BIND	New York Cognition Questionnaire	31 item scale, evaluates self-generated thoughts, 10–15 min	r = 0.18*
	Symbol Digit Coding Test (Digit Symbol Substitution Test)	Task-based, symbol-digit matching, 3–5 min	r = 0.10*
	Shifting Attention Test	Task-based, requires shifting attention, 5–10 min	r = 0.10*
	Four-Part Continuous Performance Test (CPT)	Continuous performance task, measures sustained attention, 10–20 min	r = − 0.21*
LEMON	Working Memory Trials	Task-based, involves memory tasks like n-back, 5–15 min	r = − 0.14*
	Incongruent Stimuli Reaction	Measures response time to conflicting information	r = − 0.27**

* *p < *0.05, **p < 0.005, ***p < 0.001, *ns* not significant. Abbreviations: *CAN-BIND* The Canadian Biomarker Integration Network in Depression, *LEMON* Leipzig Study for Mind–Body-Emotion Interactions

## Discussion

The present study aimed to predict placebo response in patients with MDD using a dCNN model applied to EEG data. Our model was developed using the placebo arm of the EMBARC dataset (consisting of 141 participants), with external testing performed using two independent datasets, CAN-BIND and LEMON. We demonstrated that the dCNN model based on raw EEG data achieved 69% accuracy and a ROC AUC of 0.72, highlighting a substantial ability to predict placebo responses. Analysis of the relationships between the model's placebo predictions and behavioral metadata in two independent datasets revealed significant correlations with participants’ age across all three datasets (LEMON, CAN-BIND, and EMBARC), supporting previous studies findings (Trivedi et al. 2018; MacQueen et al. 2019).

Our prediction accuracy aligns with the foundational work of Wu et al. (Wu et al. 2020) and Oakley et al. (Oakley et al. 2023). Notably, the models in these studies, as well as ours, were developed and validated using the same EMBARC dataset, reflecting the limited availability of placebo EEG data in this field. Our model, despite being more complex and less interpretable due to its deep learning architecture, achieved comparable results to previous studies. This suggests that dCNNs can effectively handle raw EEG data, reducing the need for manual feature extraction. Our work extends earlier studies (Durstewitz et al. 2018; –Krizhevsky et al. 2017) by demonstrating dCNN's proficiency in extracting complex patterns from EEG datasets without the need for extensive manual preprocessing represents considerable improvement over traditional methods. Unlike Wu et al. (Wu et al. 2020), whose predictions were significantly correlated with alpha EO condition, our regression analysis conducted on model's predictions and spectral power did not show a significant relationship with EO and EC conditions but instead highlighted associations with higher-frequency EEG components.

Our findings show both consistencies and differences with the literature regarding EEG correlates of placebo response. While previous studies(Leuchter et al. 2002;, Pizzagalli et al. 2018) emphasize the role of higher baseline theta activity in the rostral anterior cingulate cortex (rACC) as predictive of improved placebo outcomes, our results suggest that theta activity in the central electrodes (C3 and Cz) is significantly associated with placebo response, rather than in rACC. Similarly, Korb et al. (Korb et al. 2009) investigated theta current density in the rACC and medial orbitofrontal cortex (mOFC) and found that while higher baseline theta in these regions differentiated medication responders, there were no significant differences in baseline theta activity between placebo responders and non-responders. Lastly, relationships between cognitive task performance and placebo response raise the possibility that faster cognitive processing might be related to placebo response (Tétreault et al. 2016;, Leuchter et al. 2002). Specifically, the reduced beta activity in frontal areas observed in regression analyses of the model built on EMBARC may be reflectedon the LEMON and CAN-BIND datasets, where predictions demonstrated a correlation between possible placebo responders and faster reaction times. In future studies, it will be important to evaluate whether processing speed might be a proxy for prefrontal cortex function, which has been linked to placebo response.

Lower power in the frontal and central regions could also serve as a potential marker of the placebo response, in particular, when considering that frontal regions and lower beta power are linked to cognitive functions (Fodor et al. 2018), (Friedman and Robbins 2022), (Jobson et al. 2021), it is plausible that these neurophysiological measures reflect the underlying cognitive processing involved in placebo responses. For instance, Wager and Atlas (Wager and Atlas 2015) have reviewed how frontal cortical regions are implicated in placebo effects, while Koban and Wager (Koban and Wager 2016) have identified neural mediators, including frontal networks that underlie placebo analgesia. Frontal electrode sites are known to be critically involved in higher-order cognitive functions such as working memory, decision-making, and executive control. Moreover, the lower beta frequency range (typically 13–20 Hz) has been associated with top-down cognitive control and the maintenance of cognitive sets, processes essential for adaptive behavior. Previous studies have highlighted that modulations in lower beta power may indicate shifts in cognitive state and are closely linked to attentional and inhibitory mechanisms (Engel and Fries 2010), (Klimesch 1999), (Buschman and Miller 2007), (Schmidt et al. 2019), (Benedetti et al. 2007). Collectively, these findings highlight the potential role of frontal cortical regions and lower beta oscillations as neurophysiological indicators of placebo responsiveness, providing promising targets for future investigation into the cognitive mechanisms underlying placebo effects.

Further, our study explored the impact of demographic and psychological factors on placebo response, addressing points raised in the introduction about variability across different demographic groups.

The negative association between age and placebo response is consistent with prior studies (Vambheim and Flaten 2017; Weimer et al. 2015; Agarap 2018), including a prior meta-analysis by Weimer et al. (Weimer et al. 2015). The negative association between age and placebo response indicates that younger individuals may have a heightened expectancy or susceptibility to placebo effects, potentially due to fewer prior experiences with medical treatments, leading to stronger anticipatory responses (Korb et al. 2009). This goes in harmony with the previous findings by Trivedi et al. (Trivedi et al. 2018) and meta-analysis by Weimer et al. (Weimer et al. 2015), who associated younger age with heightened placebo response.

In terms of psychological factors, we investigated how personality traits may impact placebo responses. In previous studies, some of the Big Five components has been linked to potentiated placebo responses (McMillan et al. 2022), (Kern et al. 2019), (Geers et al. 2010). Specifically, previous research by Geers et al. (Geers et al. 2010; Geers et al. 2007), demonstrated that traits like extraversion, optimism, may predict greater susceptibility to placebo effects.​ Our findings complement this statement and show that indeed extraversion (EMBARC), lower self-esteem (CAN-BIND), sensation seeking and external thinking (LEMON), may predict a greater susceptibility to placebo effects. However, these findings stand in contrast to the meta-analysis by Kang et al. (Kang et al. 2022), which reported no significant association between the Big Five personality traits (extraversion, agreeableness, openness, conscientiousness, and neuroticism) and placebo response. This discrepancy may be due to differences in study designs, sample characteristics, or measurement methods for personality traits, indicating a need for standardized approaches in future research. In sum, individuals characterized by extraverted behavior, low self-esteem, and faster reaction times may be especially prone to placebo responses.

### Limitations and Potential Impact of the Current Study

The current findings offer valuable insights into the predictive modeling of placebo responses using a deep CNN. However, these results must be interpreted within the context of several limitations. A primary limitation is our indirect analysis of the deep CNN model's generalizability, which was conducted using the LEMON and CAN-BIND datasets. Notably, both datasets employed for external testing did not contain specific placebo response data, therefore containing no information that would otherwise be considered a"target"for our model. The lack of direct placebo response data in the external datasets limits the ability to fully validate our model's predictive capability. This constraint may have introduced biases or confounding factors that affect the generalizability of our findings. Also, we acknowledge inherent methodological variability between single-site studies (LEMON) and multi-site datasets (EMBARC and CAN-BIND), with multi-site studies typically introducing additional heterogeneity due to differences in recruitment strategies, EEG equipment, and recording protocols.

In our study, we applied an identical EEG preprocessing pipeline across all datasets (EMBARC, CAN-BIND, and LEMON), restricting analyses to the standard 19-channel configuration with consistent filtering, artifact correction, and segmentation protocols. Although this standardized procedure did not explicitly correct or statistically model site-specific differences, it enabled preliminary exploration of EEG-derived placebo predictions and related demographic and psychological covariates across datasets. While direct assessment of generalizability was limited due to the absence of placebo-response measures in external datasets, our findings provide initial evidence of promising transferability, aligning with the moderate cross-trial generalizability reported by Zhukovsky et al. (Zhukovsky et al. 2025) in predicting antidepressant treatment outcomes. Their study demonstrated that achieving even moderate generalizability across multi-site trials is indicative of meaningful biomarker identification despite methodological and population variability. We anticipate that future research explicitly incorporating direct placebo-response data and statistical modeling of site-specific effects will further enhance the transferability of EEG-based predictive models across diverse clinical datasets.

Further, in our current study we have included both healthy and clinical participants populations from the CAN-BIND and LEMON datasets, which prevents definitive conclusions regarding whether EEG correlates identified are specific to MDD or generalizable to placebo responses broadly. Clarifying this distinction through future studies comparing distinct clinical and non-clinical cohorts would significantly enhance interpretability.

In spite of these limitations, the current findings show substantial promise. Accurate prediction of placebo responses could assist in designing more effective and efficient clinical trials and developing personalized treatment strategies. Such advancements could not only enhance individual patient care but also have broader impacts on the efficient evaluation of novel antidepressant compounds. Our use of data augmentation techniques to address data scarcity, as mentioned in the introduction, enabled the model to learn from a broader dataset by generating additional synthetic data from existing samples.

However, the current limitations of our study, particularly the lack of direct placebo data in our testing datasets, temper these implications. Future studies should aim to validate the deep CNN model using datasets that include specific placebo response data, thereby strengthening the model's predictive accuracy and generalizability. Additionally, exploring the model's applicability across different conditions and domains could provide deeper insights into the characteristics of placebo responses that are shared across various mental health conditions. Such investigations would not only address the limitations highlighted here but also pave the way for more robust and clinically applicable predictive models.

## Supplementary Information

Below is the link to the electronic supplementary material.

Supplementary file1 (DOCX 11 KB)

Supplementary file2 (XLSX 85.7 KB)

## Data Availability

No datasets were generated or analysed during the current study.
