# Supplementary material for: Predicting Placebo Responses Using EEG and Deep Convolutional Neural Networks: Correlations with Clinical Data Across Three Independent Datasets
Source: Neuroinformatics. 2025 May 19;23(2):32. doi: 10.1007/s12021-025-09725-6 (PMC12089153; doi:10.1007/s12021-025-09725-6)
Supplement: Supplementary file 1 — Supplementary file1 (DOCX 11 KB) [file 12021_2025_9725_MOESM1_ESM.docx]

| Appendix 1. The electrode conversion table. | |
| --- | --- |
| Electrode | Label |
| E1 | f10 |
| E2 | af8 |
| E3 | af4 |
| E4 | f2 |
| E6 | fcz |
| E9 | fp2 |
| E11 | fz |
| E13 | fc1 |
| E15 | fpz |
| E16 | afz |
| E19 | f1 |
| E22 | fp1 |
| E23 | af3 |
| E24 | f3 |
| E26 | af7 |
| E27 | f5 |
| E28 | fc5 |
| E29 | fc3 |
| E30 | c1 |
| E32 | f9 |
| E33 | f7 |
| E34 | ft7 |
| E36 | c3 |
| E37 | cp1 |
| E38 | ft9 |
| E41 | c5 |
| E42 | cp3 |
| E45 | t7 |
| E46 | tp7 |
| E47 | cp5 |
| E51 | p5 |
| E52 | p3 |
| E55 | cpz |
| E57 | tp9 |
| E58 | p7 |
| E60 | p1 |
| E62 | pz |
| E64 | p9 |
| E65 | po7 |
| E67 | po3 |
| E70 | o1 |
| E72 | poz |
| E75 | oz |
| E77 | po4 |
| E83 | o2 |
| E85 | p2 |
| E87 | cp2 |
| E90 | po8 |
| E92 | p4 |
| E93 | cp4 |
| E95 | p10 |
| E96 | p8 |
| E97 | p6 |
| E98 | cp6 |
| E100 | tp10 |
| E102 | tp8 |
| E103 | c6 |
| E104 | c4 |
| E105 | c2 |
| E108 | t8 |
| E111 | fc4 |
| E112 | fc2 |
| E116 | ft8 |
| E117 | fc6 |
| E121 | ft10 |
| E122 | f8 |
| E123 | f6 |
| E124 | f4 |
| E129 | cz |
|  |  |
